# Wet Lab Techniques for the Functional Analysis of Circular RNA

**DOI:** 10.3390/cells14231920

**Published:** 2025-12-03

**Authors:** V. Praveen Chakravarthi, Lane K. Christenson

**Affiliations:** Department of Cell Biology and Physiology, University of Kansas Medical Center, 3075 HLSIC, 3901 Rainbow Blvd., Kansas City, KS 66160, USA

**Keywords:** miRNA sponge, circRNA function, protein sponge, back-splicing

## Abstract

Circular RNAs (circRNAs) emerge as alternate regulators of gene expression. CircRNAs are generated by back-splicing processes, are highly conserved, and are resistant to degradation. Recent advances in sequencing and computational tools have led to the discovery of the critical regulatory roles of these molecules in different physiological and pathological processes. Different functions of circRNAs in many physiological processes have been reported in the past few years, such as miRNA sponge activity, protein decoy/sponge/recruiter activity, deviation from parental gene expression, and encoding proteins/peptides. Additionally, circRNAs are being used clinically as biomarkers. Technological advances in molecular biology over the past few years have led to the development of various techniques for detecting, quantifying, manipulating, and analyzing the functions of circRNAs. This article summarizes different wet lab techniques for preparing, detecting, validating, localizing, and interacting with circRNAs, as well as determining miRNA sponge activity and functional analysis.

## 1. Introduction

Circular RNAs (circRNAs) are covalently closed RNAs generated by lariat-driven circularization or RNA-binding protein (RBP)-directed back-splicing [1]. Circular RNAs were first discovered in viruses, fungi, protists, plants, and finally in mammals [2,3,4,5,6]. Upon their initial discovery in 1976, circRNAs were thought to be byproducts of transcription. But subsequently, over the next five decades, direct synthesis of circRNAs over their linear parental RNA forms and functional activities have been discovered [7,8]. Evolutionarily conserved, circRNAs have been shown to regulate parental gene expression through multiple mechanisms. These mechanisms include (a) epigenetic DNA methylation [9], (b) competition with parental gene expression [10], and (c) interference with RNA polymerase II activity where the circRNA forms a complex with U1 snRNPs, thus promoting the transcription of its parental gene [11]. In addition, a well-described mechanism linked to circRNA action is their ability to bind to specific miRNAs based on miRNA seed sequence motifs, thereby acting as a sponge and decreasing miRNA availability and upregulating the expression of the miRNA target genes [12]. Like miRNA sponge activity, circRNAs can also serve as protein decoys, scaffolds, and recruiters [11,13]. Lastly, in rare cases, circRNAs have been identified with internal ribosomal entry sites (IRESs) or N6-methyladenosine-mediated cap sites, allowing them to be translated into proteins [14]. Recent work has pushed circRNAs into the scientific light as they are recognized to be important in many physiological and pathological processes. Functionally, circRNAs are known to have major roles in cellular metabolism [15,16]. CircRNAs are gaining clinical significance because of their potential to act as valuable tools for the diagnosis, prognosis, and treatment of diseases [17,18,19]. Computational approaches, sequencing strategies, techniques involved in identification, detection, and sequencing, and functional approaches have been described in some review articles either individually or in some sort of combination [20,21,22,23,24,25]. The present review compiles all the current wet lab techniques—from RNA isolation to detection, localization, and functional characterization—in a clear and illustrative manner, with the aim of serving as a comprehensive toolbox for researchers who are currently working in this field or intend to do so in the future.

## 2. Circular RNA Library Preparation and Sequencing Analysis

CircRNA library preparation and sequencing have several challenges due to low expression levels, high sequence similarities with linear RNAs, sequencing artifacts, difficulties in distinguishing true circular forms from linear contaminants, and difficulties in determining full-length sequences from short-read data. Because of low abundance, circRNA quantification is unreliable in normal RNA-seq data. For accurate quantification, it is necessary to perform deep sequencing with long reads (>100 nt). Determination of circRNA is based on back-splice junction (BSJ) identification which will not define the internal composition of circRNA. In this case nanopore-based long-read sequencing technology can help in determining the entire sequence of circRNA [20]. CircRNA enrichment helps in the identification of low-abundant or low-copy-number circRNA. CircRNA isolation strategies and circRNA enrichment are explained in Section 3.1 and Section 3.2. Further different sequencing strategies used for the enrichment and accurate profiling of circRNA are explained in this section. Sequencing artifacts can be minimized by using various bioinformatics tools including CIRCexplorer, CIRI-AS, CIRCexplorer2, and circSPlice, which are explained in Table 1, eliminating false positives.

**Figure 1:**
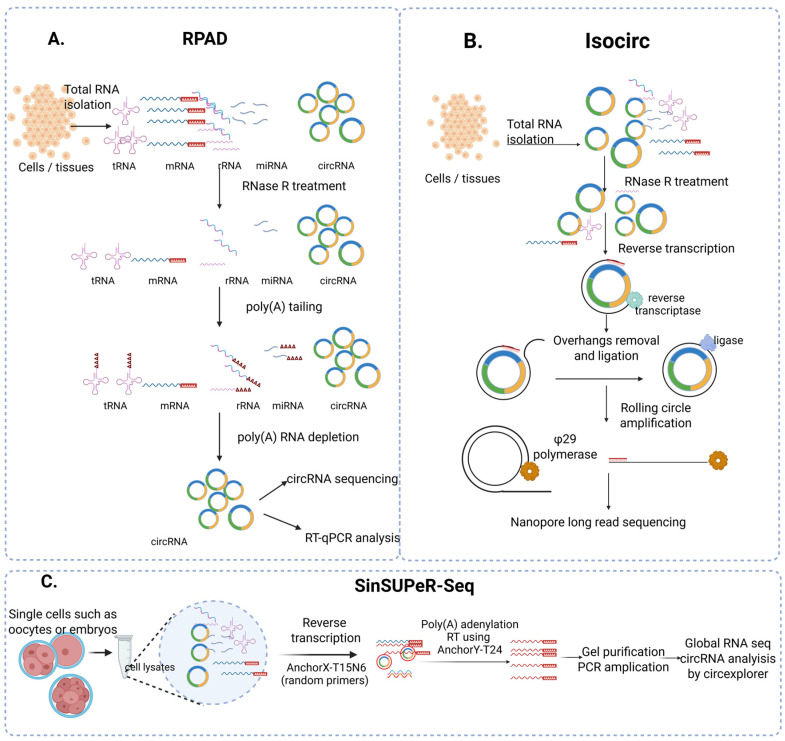
CircRNA sequencing library preparation strategies. (**A**) RPAD method uses RNase R treatment followed by poly(A) tailing and then poly(A) RNA depletion using oligo dT-Dynabeads to remove all mRNA, rRNA, and other RNA isoforms with 3′ ends, leading to enrichment of circRNA. (**B**) Isocirc method uses RNase R enzymatic digestion, reverse transcription of circRNA and ligation to create circular cDNA, followed by rolling circle amplification using Phi DNA polymerase with its strand displacement activity to generate long single-stranded DNA for nanopore sequencing. (**C**) SinSuper-Seq: Developed for single cells such as oocytes and limited cell number embryos samples are directly exposed to random primer reverse transcription followed by polyadenylation, reverse transcription using AnchorY-T24, gel purification, and PCR amplification. This technique enhances the quantity of cDNA in preparation for global RNA sequencing, followed by circRNA analysis by CIRCexplorer.

Different strategies have been developed to improve circRNA identification efficiency and reduce false positive rates. Pandey et al. [26] developed the RPAD [RNase R treatment followed by polyadenylation (poly(A)) and subsequent poly(A) RNA depletion] method to enrich highly purified circRNA from total RNA. In this method, total RNA is first treated with RNase R, removing most linear RNA and rRNA. Following RNase R treatment, the remaining RNA is poly(A) tailed. Then, these polyadenylated RNA samples are incubated with Oligo-dT Dynabeads on a magnetic stand for a short time to eliminate the remaining linear RNAs from the supernatant, resulting in an enriched pool of circRNA, either for sequencing or RT-qPCR (Figure 1A) [26]. A second methodology developed by Xin et al. [27], called isoCirc, combines rolling circle amplification (RCA) with nanopore long-read sequencing technology to characterize full-length circRNA isoforms. In brief, total RNA is first treated with RNase R, eliminating much of the linear RNA, and this is followed by reverse transcription of circRNA using random primers. 5′ hangings will be removed by nuclease digestion followed by ligation of RT products into a circular cDNA, which is then amplified by RCA. Phi29 DNA polymerase has strand displacement activity; it can displace downstream DNA strands and can continue the polymerase activity, producing a long single-stranded DNA. The resultant amplified product is subjected to nanopore long-read sequencing [27] (Figure 1B). CircRNA sequencing in single cells such as oocytes, zygotes, or 2–16 cell embryos is carried out by Single Cell Universal Poly(A)-Independent RNA sequencing (SinSuper-Seq) [28,29]. In this method, cell(s) are lysed and RNA is reverse transcribed (without RNA isolation) to generate the first strand of cDNA. A poly(A) tract is then added to the 3′ end of cDNA, followed by the synthesis of the second strand of cDNA through annealing to the poly(A) tract. These products are subjected to gel purification, PCR amplification using 5′-amine terminated primers, and then subjected to high-throughput sequencing (Figure 1C) [28,29], followed by computational approaches to identify BSJ sites. Use of computational algorithms such as Find_circ, CIRI, and CIRCexplorer has led to the detection of thousands of circRNAs across multiple species [30,31,32,33,34,35]. Further computational pipelines such as CIRCexplorer3-CLEAR, CIRIquant, and DCC can be used to quantify the relative expression of specific circRNAs by normalizing them to their corresponding linear RNAs [35]. miRspongeR, an R/Bioconductor package, and circRNA interactome can be used to analyze the miRNA sponge and protein decoy activity of circRNAs [36,37]. CircRIP is used to identify RBP and circRNA interactions from RIP-seq and eCLIP data sources [38], while CircCode, a Python 3-based program, identifies the possible coding ability of circRNAs [39]. In brief, the tools used for circRNA identification, mapping, and functional analysis are given in Table 1.

**Table 1:** Summary of tools used for circRNA analysis.

Tool	Mapper or Program	Functions	Ref.
Find-circ	Bowtie2	Detect BSJ sites to identify circRNA	[40]
CIRI	BWA		[41]
CIRCexplorer	TopHat/STAR		[42]
CIRI-AS	TopHat/STAR/MapSplice/BWA/segemehl	Identify multiple circRNAs from one gene locus. Inspect alternate circularization	[43]
CIRCexplorer2			[44]
circSPlice	STAR		[45]
CIRCexplorer3-CLER	TopHat/STAR/MapSplice/BWA/segemehl	Identify and compare circRNA with linear RNA expression	[46]
CIRIquant	BWA		[47]
DCC	STAR		[48]
miRspongeR	Bioconductor package	Identifies and analyzes miRNA sponge interactions	[36]
CircInteractome	Web-based tool	Explores circular RNAs (circRNAs) and their potential interactions with RNA-binding proteins (RBPs) and microRNAs (miRNAs)	[37]
CircRIP	Available as a command-line tool via its GitHub page	Utilizes RNA immunoprecipitation sequencing (RIP-Seq) and enhanced cross-linking immunoprecipitation (eCLIP) data to detect circRNA protein interactions	[38]
CircCode	Python 3-based pipeline	Analyzes ribosome profiling data (Ribo-Seq) and predicts whether a given circRNA can be translated	[39]

## 3. Detection and Validation of circRNA

CircRNAs are typically detected using RNA sequencing data and computational tools (Table 1 and Table 2). For confirmation and validation, appropriate orthogonal methods such as RT-qPCR, Northern blot, and in situ hybridization can be used.

### 3.1. RNA Isolation and Sample Preparation

CircRNA profiling or circRNA studies require total RNA isolation as the starting material [20]. Different methods of RNA isolation can be used for circRNA profiling, such as TRIzol, GENExol, RNeasy kit, MagMaX, and MiniPrep. TRIzol method is widely used, efficient, yields high concentration, and can be used for various samples, including tissues, cells, and fluids [49,50]. TRIzol method was found to be optimum for the isolation of RNA from fluid samples such as human urine sediment [51]. But yields with TRIzol method have contaminants such as DNA, proteins, and PCR inhibitors [49,50]. MagMAX or miniprep kits produce less yield compared to TRIzol method [49,52]. TRIzol method needs a large amount of sample to produce a good yield of RNA; if the sample amount is less, proceeding with kits such as RNeasy or MagMax or MiniPrep is best [49,52]. Tesena et al. [52] reported GENEzol as the best method in terms of yield and purification, and it is cost-effective compared to TRIzol or the MiniPrep method.

### 3.2. circRNA Enrichment Strategies

Since most circRNAs are expressed at lower levels and are difficult to analyze compared to linear RNAs; rRNA removal, RNase R treatment, and poly(A) counterselection are used to enrich for circRNA. Different methods for rRNA depletion are available, such as oligonucleotide hybridization to rRNA, pulldown with hybrid-specific antibodies attached to magnetic beads [53], commercial kits (RiboZero Magnetic Gold kit, Epicentre, # MRZG12324, Madison, WI, USA) [54], or hybridization of rRNAs with ssDNA probes followed by RNase H digestion, referred to as the rRRR method [55]. RNase free rRNA-depleted samples are useful for the simultaneous detection of circRNAs and their corresponding linear RNAs. In contrast, circRNA enrichment using RNase R or poly(A) counter selection methods which enhance the detection of circRNA, prevents the simultaneous analysis of both circRNA and its corresponding linear RNA [20]. RNase R treatment degrades most linear RNAs, enhancing by as much as 10-fold the level of circRNA, cANRIL in an RNase R-treated sample compared to the untreated sample [56,57]. However, some circRNAs, such as circPan and circAnk2, are known to be sensitive to RNase R treatment [4]. Poly(A) selection also depletes some circRNA species, leaving their counterpart mRNAs [4]. Choosing the optimum method of circRNA enrichment is based on the requirements, such as the circRNA vs. linear RNA ratio or just the circRNA differential expression.

### 3.3. RT-qPCR

**Figure 2:**
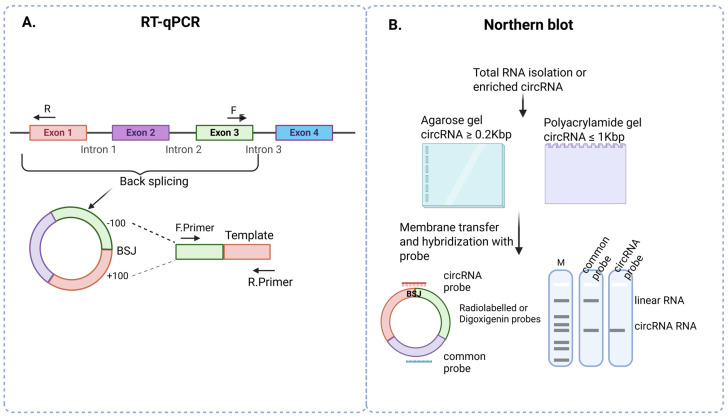
Detection and validation of circRNA. (**A**) RT-qPCR: circRNA RT-qPCR involves template design using 100 bp upstream and 100 bp downstream of the back-splicing junction. This template is used for primer design, and quantitative analysis can use either TaqMan probe or SYBR Green based detection methods. (**B**) Northern blot: Northern blot analysis of circRNA involves either agarose gel electrophoresis (size ≥ 0.2 kb) or polyacrylamide gels (size ≤ 1 kb). Radiolabeled or digoxigenin probes designed to span the BSJ, and a common probe designed to detect another exon, will allow distinction of the linear and circular forms based on their size in Northern blot analysis.

RT-qPCR is a commonly used technique for the detection and validation of circRNA [6,58,59,60]. Divergent primers spanning the BSJ amplify the specific circRNA, avoiding linear RNA. Briefly, a PCR template is constructed by joining a 100-nucleotide sequence downstream of the 5′ end with a 100-nucleotide sequence upstream of the 3′ end of the BSJ for primer design [6,58,59,60]. Primers that overlap the junction site can be used, but with care to ensure that the intronic sequences present in the nascent RNA are not like those created at the BSJ site. In the context of simultaneous evaluation of circRNA and linear RNA, DNase treatment of the isolated RNA is of value to ensure that the primers for the linear RNA are not amplifying cellular DNA. In this context, for the linear RNA, a no-RT control is of value. Consistent with normal RT-qPCR for linear targets, circRNA RT-qPCR products should show a single product in agarose gel or a single peak in temperature dissociation analyses (Figure 2A). Instead of the SYBR Green method, the use of a TaqMan probe provides even more sensitivity and accuracy for circRNA detection. The crucial part of this method is designing the TaqMan probe to specifically target the BSJ sequence, which will eliminate the detection of linear RNA and enable the specific detection of circRNA [61]. The SYBR Green method is a more flexible, cost-effective method and is particularly useful in the initial screening process. Similar to mRNA analyses an additional melt curve analysis at the completion of PCR should be completed to verify specificity. In conclusion, depending on whether the investigation is solely interested in circRNA expression or if understanding the diversion of the linear RNA form to the circRNA is of interest, the methods for quantitation and validation of circRNA can vary as described above.

### 3.4. Northern Blot

Northern blot is a direct RNA-based method that does not require reverse transcription or PCR but does rely on the availability of much higher concentrations of RNA. In those cases where a BSJ is present between distant exons, the Northern blot can provide detailed information regarding the size (inclusion of other exons) and circular conformation based on the migration of bands. A specific probe designed against the BSJ sites will detect circRNA, while a common probe (preferably within the same exon as one of the BSJ exons) will allow for detection of circRNA with variant numbers of included exons as well as the linear form. Limitations of Northern blot include the requirement of a large amount of sample, low sensitivity, and the inability to accurately quantify the levels of circRNA compared to linear RNA. Typical Northern blot uses either agarose gel (1.2%) or polyacrylamide gel. For the detection of circRNA from 0.2 Kbp to several Kbp, agarose gels are suitable. For the detection of circRNA with size ≥ 1 Kbp, polyacrylamide gels are best and allow a clear distinction between circRNA and linear RNA forms based on different mobilities. Radiolabeled or newer digoxigenin probes, which are more sensitive, safer to handle (i.e., non-radioactive), and provide higher resolution, are used for detection [62,63] (Figure 2B). Though time-consuming and with several limitations, Northern blots do provide a valuable and informative approach, as shown for circRNA such as CircRHOT1 [64], circSMARCA5 [65], circPTK2 [66], and recatch [67].

### 3.5. In Situ Hybridization

**Figure 3:**
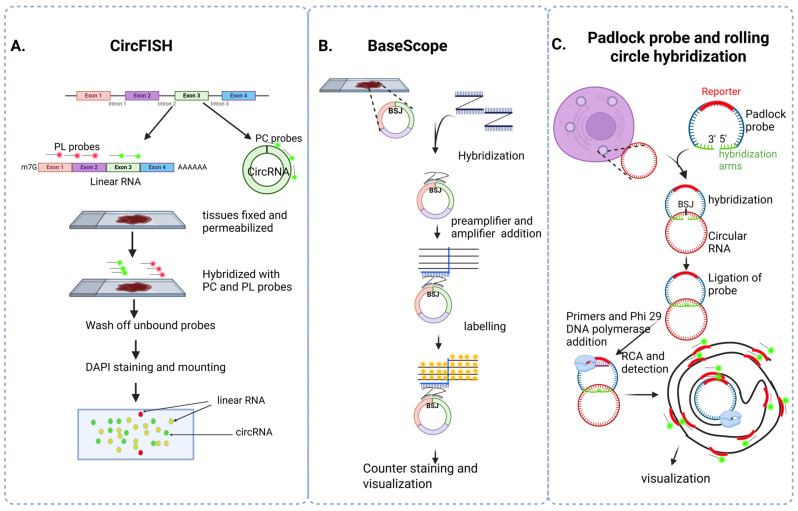
Visualization or localization of circRNA. (**A**) CircFISH: CircFISH uses probes specific to linear RNA (PL probes) and circRNA (PC probes) with different attached fluorophores. Merging the two images will result in the detection of circRNA (green color) and linear RNA (yellow and red). (**B**) BaseScope: BaseScope in situ hybridization involves the binding of Z-stack pair probes on either side of the BSJ, followed by branch chain amplification with preamplifiers and amplifiers to visualize low-copy-number circRNA. (**C**) Padlock probe and rolling circle hybridization: Padlock probes, which consist of a reporter sequence and two circRNA hybridization arms that bind to opposite sides of the BSJ, are followed by the addition of primers and Phi 29 DNA polymerase, which results in rolling circular amplification. Probes specific to the reporter sequence are then incorporated to allow the detection and visualization of circRNA.

The precise function of circRNA depends on its subcellular localization, and RNA fluorescent in situ hybridization (RNA FISH) typical for linear RNA localization often requires slight modifications for circRNA [68]. The major limitations in detecting circRNA by RNA FISH are its lower copy number compared to their linear counterparts and interference from linear RNA during detection. To overcome these limitations, alternative strategies have been developed, three of which are shown in Figure 3.

CircFISH involves using two different probe sets; the first probe set was named as probe circular (PC), specifically for circRNA. The second probe set was named as probe linear (PL), which binds to exons in the linear RNA, which is not a part of circRNA. The PC probe set will be labeled with Cy5 (green) and PL probes with Texas red. CircRNA has a binding site only for the PC probe set, so its fluorescence is green. Both PC and PL probes bind to linear RNA, and the fluorescence of both probes merges and exhibits a yellow color [68] (Figure 3A).

BaseScope, a unique FISH system, uses Z probe pairs that contain an 18–25 bp complementary sequence to the target sequence (BSJ), followed by a spacer sequence and a short-tailed sequence, which are recognized by the signal amplification system. Z probe pairs bind on either side of the back-splicing junction. Upon the addition of preamplifiers, preamplifiers will bind to the tail sequence of Z pair probes only when both Z pair probes are in close vicinity, which allows the detection of just circRNA. Each preamplifier will bind to many amplifier molecules. Each amplifier molecule has many binding sites for labeled probes. Thus, preamplifiers, amplifiers, and finally, many probes will boost the signal [69,70] (Figure 3B). BaseScope can use fluorescent probes or chromogenic enzymes, followed by substrate inclusion, and has been used for the detection of circSamd4 [71], circSHKBP1 [72], circAR3 [73], circPLEKHM3 [74], circSlc45a4 [75], circCACNA2D1 [76], and circCACNA1E [76] in different tissues.

A third alternative and simple method for the detection of circRNA is based on the use of padlock probes, which consist of two short nucleotide sequences, 15–20 bp in length, complementary to the BSJ on either side, and a reporter signal. After hybridization, DNA ligation is carried out to join the head and tail of the padlock probes, followed by RCA with a primer complementary to the padlock probes. RCA is an isothermal method for exponential amplification of circular DNA or RNA. RCA uses a special DNA polymerase, most commonly Phi29 DNA polymerase, which can extend the primer around the circular template. It also has strand displacement activity, which means it can displace or push aside any downstream DNA strands it encounters and continue the polymerase activity, producing long single-stranded DNA containing many repeated copies of original template sequences with many reporter sequences. Fluorescently labeled probes complementary to the rolling circle amplification products (RCPs) are applied to hybridize and detect the RCPs. This allows even low-copy-number circRNA to be visualized with high efficiency [77] (Figure 3C).

## 4. Identification of RBPs Involved in circRNA Biogenesis

Once a circRNA’s presence is established in specific tissues or cells of interest, the next focus may be on the regulation of its expression. Biogenesis of circRNA is facilitated by RNA-binding proteins (RBPs), which influence back-splicing events and circularization efficiency. RBPs act as either activators or inhibitors of circRNA biogenesis. RBPs bind to RNA via RNA-binding domains (RBDs) [78,79] to promote circRNA biogenesis by dimerization, which connects the upstream and downstream splicing sites and promotes the back-splicing event [80,81,82]. RBPs can also inhibit the circRNA biogenesis either by disrupting the Alu repetitive sequence [83,84] or by binding to the reverse complementary sequence [85] located in flanking regions of the BSJ.

To identify RBPs involved in circRNA biogenesis, tissue-specific RBP expression under different physiological conditions or following treatments is typically employed [86]. Different RBPs involved in the biogenesis of circRNA in a wide range of tissues under different physiological conditions have been reported in the past decade [5,10,87]. In 2022, a circRNA regulator identification tool (CRIT) was developed to identify circRNAs that interact with a group of 73 RBPs [88]. These 73 novel regulators of circRNA biogenesis were identified by annotating thousands of samples of cross-linked immunoprecipitation results combined with short interfering RNA-(siRNA) or short hairpin RNA (shRNA)-mediated knockdown of RBPs [88]. These 73 RBPs can be used to screen circRNA biogenesis in different tissues or cell types. CircRNA–RBP interaction can be predicted using different computational tools such as CRIP, circSLNN, DeCban, RPISEq, RPI-Pred, and RBPmap [89,90,91,92,93,94]. These tools can be used to screen the RBP interaction in the vicinity (upstream or downstream) of the BSJ, which can be used to predict the role of RBP in circRNA biogenesis. For further validation or confirmation of the RBP role in circRNA biogenesis, RNA immunoprecipitation (RIP) or cross-linking immunoprecipitation (CLIP) can be used. Usually, RIP or CLIP experiments are used to identify the interaction of RBP with circRNA. The role of RBP in circRNA biogenesis can be detected by screening the interaction of RBP with circRNA near the vicinity of the BSJ, where the binding of RBPs can induce the joining of upstream and downstream RNA to form circRNA upon dimerization.

### RNA Immunoprecipitation (RIP)

RIP can be used to study the interaction between RNA and protein, typically RNA-binding proteins. This principle was exploited to study the role of RBP in circRNA biogenesis as well as the sponging activity of circRNA.

**Figure 4:**
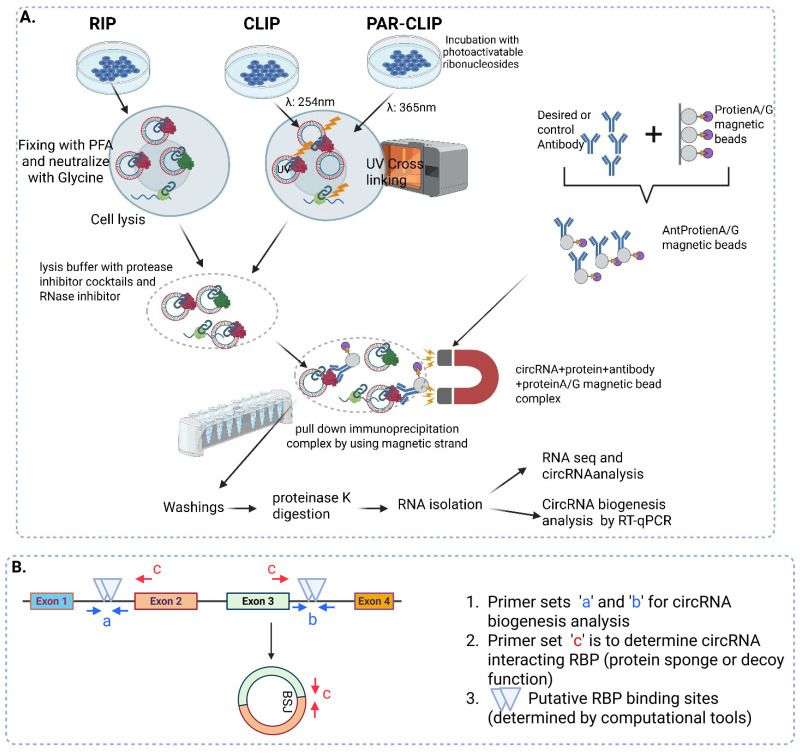
RIP, CLIP, and PAR-CLIP: (**A**) RNA immunoprecipitation involves fixing RNA and protein interactions using formaldehyde-based solutions, followed by neutralization with excess glycine, whereas CLIP involves cross-linking RNA and protein via ultraviolet radiation at 254 nm, modifying the RNA to bind nearby proteins. In the PAR-CLIP protocol, cell lysates are incubated with photoactivatable ribonucleosides and exposed to UV light at the 365 nm range. In all three assays, after the cross-linking procedure, antibodies against specific RBPs saturated with protein A/G magnetic beads are used to pull down the RNA–protein complex. Magnetically separated circRNA–protein complexes are then subjected to proteinase K digestion followed by RNA isolation. (**B**) For detecting the role of RBP in circRNA biogenesis, primers ‘a’ and ‘b’ designed against the putative binding sites near the vicinity of the BSJ are used. For detecting the protein decoy or protein sponge activity of circRNA against the RBP, primers ‘c’ spanning the BSJ are useful.

In brief, the RIP protocol involves formaldehyde fixation of cells followed by quenching with glycine, which preserves the cross-linking between RNA and proteins. Antibodies against the desired RBP will be saturated with protein A/G magnetic beads and incubated with cell lysates. Pulldown RNA-RBP complexes will be treated with proteinase K for the digestion of proteins, and the residual sample will be used for RNA isolation (Figure 4). For detecting the role of RBP in circRNA biogenesis, primers designed against probable binding sites (as determined from computational tools such as CRIP, circSLNN, DeCban, RPISeq, RPI-Pred, and RBPmap) in the vicinity of the BSJ, which favor circRNA formation, will be used to quantify the binding affinity of RBP. The role of Quaking (QKI) in the biogenesis of circSMARCA5 was evaluated by RIP assay, where qRT-PCR was used to detect the QKI occupancy within the introns adjacent to the circRNA-forming exons. This study showed that QKI binds to the previously validated QKI target sites near the exon-adjacent sites, which leads to the formation of circRNA, and the binding to other regions was found to be negligible [87]. RBPs can also inhibit circRNA biogenesis in some cases; for example, in DLC1 RNA, reverse complementary sequences between introns 13 and 16 (I13RC and I16RC) favor the formation of circDLC1. DHX9 suppression leads to a significant increase in the expression of circDLC1. RIP assay with DHX9 antibody showed high binding probability of DHX9 at I13RC and I16RC in hepatocellular carcinoma cells. Thus, DHX9 binding at I13RC and I16RC prevents the circularization and formation of circDLC1 [85].

While formaldehyde preserves the protein–protein and RNA–protein interactions, there are chances that it may also capture circRNAs bound to other, non-specific proteins (Figure 4). To overcome this error and to increase specificity, a variant of RIP called cross-linking immunoprecipitation (CLIP) was developed, where ultraviolet light (UV) was used to create cross-linking between RBPs and single-stranded RNA (ssRNA). UV light stimulates the formation of covalent bonds between RBPs and directly bound RNAs, unlike formaldehyde, which promotes protein–protein and RNA–protein interaction [95] (Figure 4). In CLIP, UV cross-linking is performed at 254 nm. In a variant of CLIP, called PAR-CLIP, UV cross-linking is performed at 365 nm after incubating the cells with photoactivatable ribonucleosides such as 4-thiouridine (4SU) or 6-thioguanosine (6SG), which increases the efficiency of some RBPs and in cases of nascent RNAs [96]. AGO2 CLIP was performed to identify the interacting circRNAs with AGO2 [11].

## 5. Detecting circRNA-Interacting Partners

Differentially expressed circRNAs and their abundance and localization will be determined by RNA-seq, circRNA analysis, and orthogonal methods such as RT-qPCR, Northern blot, and in situ hybridization (Table 2). Once circRNA are identified in specific tissues or cells of interest and methods of biogenesis uncovered, the next approach may be to determine the mechanism of action of these circRNAs. Detecting circRNA-interacting partners helps in determining the two major mechanisms of action of circRNA, i.e., miRNA sponge activity or protein sponge activity.

Direct actions of circRNA typically occur via one ofthe two major mechanisms, i.e., acting as miRNA sponges or binding to proteins to sequester and regulate them. CircInteractome, miRsponge, and circRIP will give some predictions on miRNA sponge, protein sponge, or protein decoy activity of a circRNA. For further confirmation, assays such as antisense oligomer-based pull-down assay, biotin-coupled miRNA capture assay, and RIP or CLIP assays can be used. Antisense oligomer-based pull-down assay uses circRNA as the starting material, and probes designed against the BSJ are used to pull down circRNA and its interacting partners. This assay can be used to detect circRNA-interacting partners such as miRNA and proteins. RIP/CLIP/PAR-CLIP can also be used to detect circRNA interactions apart from circRNA biogenesis, where primers are designed against the BSJ in this case. RIP/CLIP/PAR-CLIP uses RBP as the starting material, and antibodies against RBPs are used to pull down RBP and its interacting circRNA. Thus, RIP or CLIP cross-verifies the interaction compared to the antisense-based pull-down assay. Biotin-coupled miRNA capture assay is used to detect the miRNA sponge activity of circRNA. In this assay, miRNA is used as the starting material, and biotinylated miRNA mimics are used to pull down circRNAs.

**Table 2:** Summary of all wet lab techniques used for circRNA analysis.

Technique	Input	Principle and Advantages	Disadvantages/ Limitations
CircRNA library preparation and sequencing strategies			
RPAD	Total RNA	CircRNA enrichment by - RNase R - PolyA selection and depletion High purity Sensitivity: high, limiting background linear RNA noise	Requires large amount of sample Multiple steps, labor-intensive, Cannot determine the circRNA to linear RNA ratio Not feasible for single cells
Isocirc	Total RNA	CircRNA enrichment by - RNase R Amplification of circRNA by RCA Even small samples are enough due to RCA Full length sequencing: determines entire circRNA sequence Sensitivity: moderate.	Higher cost Low throughput and high error rates Not feasible for single cells Cannot determine the circRNA to linear RNA ratio
SinSuper-Seq	Single cells (oocytes, embryos)	It is feasible for single cells Can determine the circRNA to linear RNA ratio	No circRNA enrichment
Detection and Validation of CircRNAs			
RT-qPCR	cDNA	Quantitative, uses divergent primers Sensitivity: high, can detect low-abundant circRNA Requires small samples Cost effective	Cannot determine localization Artifacts: overestimation No information on transcript size
Northern Blot	Total RNA	No reverse transcription or PCR is required. Uses gel electrophoresis - Agarose gel: 0.2 Kbp to several Kbp - Polyacrylamide gel: ≥1 Kbp Transcript size and isoforms can be determined Distinguishes between circRNA and linear RNA	Time consuming Labor-intensive Sensitivity: low Requires large amount of sample Quantitative expressions cannot be determined
In situ hybridization	Fixed tissues	Localization, tissues, or cell-specific expression can be determined	No information on transcript size Time consuming, Requires specialized equipment
Comparison of different strategies in in situ hybridization			
	CircFISH	Two sets of probes (PC and PL) help in simultaneous detection of both linear and circRNA as well as localization. Relatively less expensive and similar to that of standard FISH Compatible with PFA-fixed and fresh frozen tissues Sensitivity: high, offers single molecule sensitivity	Probe design and simultaneous analysis of circRNA and linear RNA is complicated
	BaseScope	Uses ZZ probes targeting BSJ High specificity Sensitivity: high due to signal amplification	High cost and labor-intensive Lower sensitivity compared to RCA
	Padlock probe and rolling circle hybridization	Uses padlock probes and RCA High sensitivity compared to Basescope High specificity: due to specific ligation and recognition.	Multiple steps Labor-intensive High cost Background interference in some cases
Identification of RBPs involved in circRNA biogenesis			
RIP	Cells or tissue lysates	Formaldehyde fixation followed by glycine neutralization Fixes RNA–protein and protein–RNA interactions Sensitivity: high sensitivity, captures low affinity RBP-circRNA interactions	Low specificity, more background noise High signal to noise ratio.
CLIP	Cells or tissue lysates	UV-C irradiation Fixes RNA–protein interaction only Sensitivity: moderate Specificity: high	Low sensitivity for some RBPs Need additional equipment (UV cross linker)
PAR-CLIP	Cells or tissue lysates	UV-A irradiation (365 nm) 4SU/6SG nucleoside incorporation Fixes RNA–protein interaction only High sensitivity Specificity: high	Requirement of live cells for nucleoside incorporation and is toxic More steps and need additional equipment
Detecting circRNA-interacting partners			
Antisense oligomer-based pulldown assay	Cells or tissue lysates	Assay is based on specific base pairing with BSJ sequence and affinity purification Specificity: targets circRNA specifically, leaving linear RNA This assay, coupled with mass spectrometry, helps in the identification of unknown protein interactions Both protein and miRNA interactions can be determined Used for the validation of circRNA interacting partners identified by computational approaches	Some non-specific binding chances Probe efficiency issues: results in low pull-down potential
Biotin-coupled miRNA capture assay	Live cells	Biotin-labeled miRNA mimics are transfected into cells and affinity-purified by streptavidin beads Direct experimental validation of circRNA- miRNA interaction High specificity: due to biotin–streptavidin affinity Resultant complex can be used for RT-qPCR or RNA seq	Needs live cells Multiple steps Labor-intensive Chances of RNA degradation are high
Functional analysis of circRNA: Overexpression of circRNA			
Plasmids	Live cells/animals	Uses commercial plasmid constructs Simple and cost-effective process	Low efficiency and short-term expression
Viral Vectors	Live cells/animals	Uses engineered viruses High efficiency Long-term expression	Integrates into the host genome, and the mutagenic risk is high Limited capacity for the circRNA size of interest
Transposon-based system	Live cells/animals	Uses cut and paste mechanism to insert the circRNA into host cells Integration in the host genome Stable and long-term expression More cost-effective than viral vectors	Potential for off-target insertion and mutagenesis
Functional analysis of circRNA: CircRNA knockdown or knockout			
SiRNA	Live cells/animals	Synthetic double-stranded RNA molecules (20–25 bp) Incorporates into the RISC and cleaves the circRNA transcript Target: CircRNA transcript Transient knockdown	Requires repeated administration for long-term studies Off-target effects are high Potentially toxic
ShRNA	Live cells/animals	Small hairpin RNA, expressed from viral vectors or plasmids Processed into siRNA within the cell Target: CircRNA transcript Transient knockdown	Stable integration and does not require repeated administration Off-target effects Potentially toxic
CRISPR–Cas13	Live cells/animals	RNA-guided RNA endonucleases Targets and cleaves circRNA Target: CircRNA transcript Transient knockdown	Non-specific cleavage of linear RNA may sometimes be toxic
CRISPR–Cas9	Live cells/animals	Cas9 is a DNA endonuclease guided by single guide RNA (sg RNA) Introduces a double-stranded DNA break leading to gene knockout Target: CircRNA gene (genomic DNA) Transient knockdown	Also affects linear RNA Permanent change in the DNA can be lethal, if the target gene is essential.

### 5.1. Antisense Oligomer-Based Pulldown Assay

**Figure 5:**
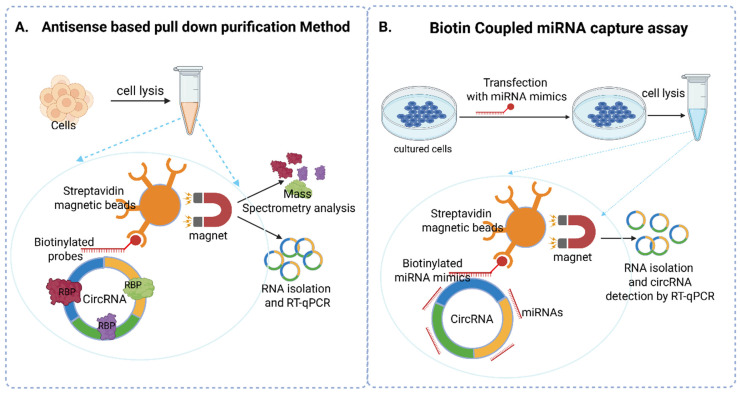
(**A**) Antisense-based pull-down purification method. The biotinylated probe spanning the BSJ of a specific circRNA can be used to pull down circRNA along with its interacting partners from cell lysates. This pull-down complex is used to detect circRNA-interacting proteins by mass spectrometry or Western blot analysis. (**B**) Biotin-coupled miRNA capture assay. Biotinylated miRNA mimics can be used to pull down miRNA-interacting circRNA. This pull-down complex is used for the isolation of RNA and determining the miRNA sponge activity of circRNA. RBP: RNA-binding protein.

The principle of this assay is based on designing antisense biotinylated oligomer probes against a specific circRNA of interest to pull down the circRNA–protein complex. The probes are designed to be complementary to the BSJ of a specific circRNA. The biotin-–streptavidin affinity principle is used to pull down the circRNA of interest. Biotinylated probes complementary to the BSJ of circRNA will be used to bind the circRNA. Streptavidin-coupled Dynabeads will be used to pull down the circRNA and its interacting partners. Biotinylated oligomers with randomized sequences will be used as controls (e.g., GCTGGTAGAGGGAGCAGATG). This pull-down sample can be used both for RNA and protein analysis [58,97,98] (Figure 5). This assay can be used for multiple purposes: (A) Proteins from this complex can be subjected to mass spectrometry to identify an array of proteins interacting with a specific circRNA, or if some information is known about interacting proteins based on CircInteractome or other tools, Western blot with antibodies against those specific proteins can be performed; (B) RNA isolated from this pull-down complex can be used for verification of the assay, i.e., by performing RT-qPCR against the circRNA on which the assay was designed; (C) RNA isolated from this pull-down complex can be used for small RNA sequencing followed by miRNA analysis. Thus, this assay can be used to determine the mechanism of action of circRNA, such as RBP interactions or miRNA sponge activity.

### 5.2. Biotin-Coupled miRNA Capture Assay

The principle of this assay is to design biotin-coupled miRNA mimics that can bind to the circRNA of interest, which can then be pulled out by streptavidin-coupled Dynabeads. The resulting complex can be washed down and used for isolation of RNA and characterization using RT-qPCR or RNA sequencing. In detail, the cells can be transfected with biotin-coupled miRNA mimics or control biotin RNAs. Twenty-four hours after transfection, the cells can be pelleted out by centrifugation and subjected to cell lysis using lysis buffer. Initially, streptavidin-coated magnetic beads can be washed with lysis buffer and incubated with cell lysates for 4 h or overnight. The pull-down complex can be washed five times with lysis buffer. The RNA bound to the beads can be isolated using TRIzol reagent. The levels of circRNA can be quantified by qRT-PCR. The enrichment ratio of the control-normalized pull-down RNA to the control-normalized input levels can be used to determine the miRNA sponging potency of circRNA (Figure 5) [67,99,100,101,102,103].

## 6. Functional Analysis of circRNA

Following detection, localization, and method of biogenesis (if of interest) and mechanism of action via which a particular circRNA may work as explained above, the next steps is to determine the actual role of a specific circRNA may have in cellular physiology using gain of function or loss of function approaches (Table 2).

Gain of function or loss of function approaches of circRNA are performed using circRNA expression plasmids or RNA interference-based strategies, respectively.

### 6.1. Overexpression of circRNA

**Figure 6:**
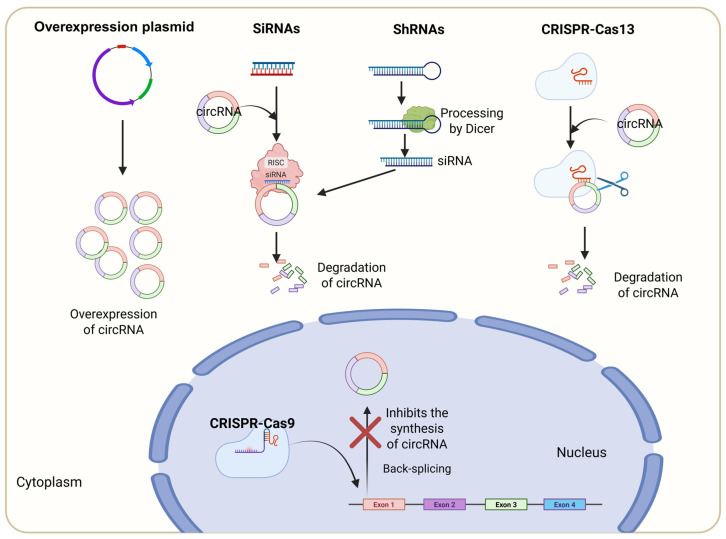
Functional analysis of circRNA. Overexpression of circRNA is achieved by viral vectors, plasmids, or transposon-based systems. siRNAs targeting the BSJ of circRNA form a RISC, which typically binds to the circRNA and degrades it. shRNAs are long hairpin loop structures that are processed by DICER to produce siRNAs. Both siRNA and shRNA derived siRNAs target and degrade the circRNA. CRISPR/Cas9 targets the intronic region with inverted complementary sequences and helps in the downregulation of circRNA synthesis, leaving linear RNA unaffected. CRISPR/Cas13 consists of RNA-guided RNase Cas13, which recognizes the BSJ of circRNA and degrades it.

circRNA constructs cloned into viral vectors or plasmids or transposon-based systems can be transferred to cultured cells to study the effect of overexpression of circRNA (Figure 6). The optimal size range for overexpression of circRNA is 100 nt–5 kb [104]. Overexpression of circPOFUT1 was achieved in gastric cancer cells using the pcDNA3.1(+) circRNA Mini Vector and transfected using Lipofectamine 3000. This resulted in enhanced autophagy-associated chemoresistance in the cancer cells, which was inhibited by overexpression of miR-488-3p [105]. circITCH overexpression adenoviral constructs transduced into hiPSC-CM cells induced significantly high levels of circITCH expression that worked as a novel therapeutic target for doxorubicin-induced cardiotoxicity [106]. Functional analysis of circRNA in prostate cancer cells was determined by transfecting 1.2 μg of circRNA overexpressing plasmids or control empty vectors by Lipofectamine 3000-mediated transfection [107]. circRNF13 was overexpressed using the pCirc (circular RNA overexpression plasmid) and transfected into nasopharyngeal carcinoma cells, which resulted in inhibition of cell proliferation [108].

### 6.2. siRNA-Mediated Depletion of circRNA

Similarly to mRNA encoding genes, circRNA function can be evaluated by siRNA depletion. Upon transfection, siRNAs are directly incorporated into the RNA-induced silencing complex (RISC) and mediate the degradation of targeted mRNA or circRNA [109]. SiRNA-mediated knockdown of circRNA expression is the most common way to reduce the expression of circRNA. The key concept here is that siRNAs should be designed against the BSJ, which will target circRNA, leaving its corresponding linear RNA untouched. But there is some risk associated with off-target effects, which can be reduced by designing two or three siRNAs at slightly different spots, still targeting the BSJ. A scrambled siRNA control sequence should be used as a control (Figure 6). Different modes of transfection of siRNA are currently being used. In oocytes and other single cells, direct microinjection of siRNAs that target circRNA can be performed, and the resultant effect of circRNA knockdown can be evaluated by quantifying the in vitro maturation and fertilization and embryo developmental potential of oocytes [110]. Lipofectamine-mediated direct transfection of siRNAs against circTau resulted in the reduction in circTau expression, fulfilling six of eight criteria such as 30–52% GC content, at least three or more A/U nucleotides in the 3′ end, an A at the 19th and 3rd positions, a U at the 10th position, no G or C at the 19th position, G at the 13th position, and no internal repeats [111]. Also, Lipofectamine 3000 was used for the direct transfer of siRNAs targeting circHIPK3, which resulted in a significant reduction in circHIPK3, affecting cell viability and proliferation in primary cultured human lens epithelial cells [112]. Thirty picomoles of siRNA or control siRNA targeting key circRNAs in prostate tumors were transfected into 3 × 10^5^ cells using 9 μL of Lipofectamine RNAiMax in Opti-MEM with reduced serum, which resulted in a significant reduction in the circRNA [107]. Transfection of cells with siRNAs targeting circ_63706 resulted in circ_63706 depletion. Implantation of circ_63706 knockdown cells into the cerebellum of NOD SCID mice resulted in small medulloblastoma tumors and a longer life span compared to parental cell implantation [113].

### 6.3. shRNA-Mediated Depletion of circRNA

shRNAs are long hairpin structures that are processed into siRNA within the cell by an enzyme called Dicer and are then incorporated into the RISC to silence genes [109]. shRNAs are often delivered using viral vectors, which induce continuous and stable expression, providing more efficient and long-term gene silencing [114] (Figure 6). Different strategies have been used for transferring shRNA into the host system to suppress circRNA. shRNA constructs targeted against circCamk1, circOgt, circPkn, and circPlexA were ligated into the Valium20 vector and were used to generate circRNA knockdown flies [115]. Cre-dependent circMET shRNAs were injected intravenously into mice, which decreased the circMET in endothelial cells [116]. Functional analysis of a set of circRNA in prostate tumors was determined by targeting circular RNA using shRNA. Lentiviral constructs of shRNA targeting circRNAs were made using the pLKO.1 vector and transfected together with packaging (psPAX2) and envelope plasmid (pMD2.G) by using Lipofectamine 3000 in prostate cancer cell lines [107]. shRNA targeting circHIPK3 delivered through the intratracheal route significantly reduced the expression of circHIPK3 and resulted in the transition of fibroblasts to myofibroblasts [117].

### 6.4. CRISPR–Cas9-Induced circRNA Knockout/Knockdown

CRISPR (Clustered Regularly Interspaced Short Palindromic Repeats)/Cas9 system comprises a guide RNA and an enzyme, Cas9, which helps cleave DNA. Guide RNA (gRNA) consists of two parts: the first part is a crRNA that binds to target DNA, and the second part is a tracrRNA that binds to Cas9. Thus, gRNA recognizes the target sequences, and Cas9 induces a blunt double-stranded DNA break, which can be repaired by nonhomologous end joining or homologous recombination (Figure 6). The Cdr1as gene has high efficiency of circularization, and usually linear transcripts are not detected, so the CRISPR–Cas9 system was used to delete the locus of Cdr1as, which eliminated circCdr1as [118]. To preserve the linear RNA expression and selectively knock down circRNA, a different strategy was followed, where the intronic regions with an inverted complementary sequence of GCN1L1 gene were targeted with the CRISPR–Cas9 system, which resulted in the selective downregulation of circGCN1L1 [119]. Another strategy was developed to edit only the bases at the back-splicing sites that prevent the biogenesis of circRNA. In this strategy, different nucleobase deaminases were integrated with the CRISPR–Cas9 system to edit C-to-T or A-to-G editing at the back-splicing sites. Successful elimination of circCDR1as/ciRS-7 was achieved by the base pair editing mechanism in 293FT cells [120].

### 6.5. CRISPR–Cas13-Induced circRNA Knockdown

Unlike the CRISPR–Cas9 system, CRISPR–Cas13 can recognize and cleave RNA. CRISPR–Cas13 comprises RNA-guided RNase Cas13 and a guide RNA with 64–66 nt that recognizes target RNA. There are three variants of Cas13, i.e., Cas13a, Cas13b, and Cas13d. Cas13a and Cas13b bind to target RNA but cause nonspecific cleavages in the adjacent RNA molecules. Cas13d is more specific and only cleaves RNA molecules guided by the specific RNA sequence. Cas13d is relatively small, easy for delivery, and can be used for RNA knockdown or gene editing [121] (Figure 6). CRISPR–RfxCas13d was used to knock down circFAM120A and circHIPK3 in HT29, HeLa, and 293FT cells using lentiviral vector constructs. Knockdown efficiency was high compared to shRNA, and the expression of corresponding linear RNAs was not affected [122]. Li et al. [122] also succeeded in microinjection-mediated transfer of CRISPR–RfxCas13d to knock down circMan1a2 and found that circMan1a2 plays a regulatory role in the development of the embryo. The CRISPR–RfxCas13d system was utilized to knock down Adar-regulated circular RNAs, i.e., circCHEK2, circGALK2, circMKLN1, circRHOT1, and circSLC39A8 in cancer cell lines EC109 and SNU398. Depletion of circCHEK2, circGALK2, and circSLC39A8 resulted in the reduction in tumorigenicity in the cells, showing that these circRNAs play a role in inducing tumors [123].

### 6.6. Assays for the Functional Analysis of the circRNA

Assays are designed based on the predicted function of a specific circRNA. Some basic assays, such as cell viability and cell proliferation assays, are useful in many cases. 3-(4,5-dimethylthiazol-2-yl)-2,5-diphenyltetrazolium bromide assay (MTT assay) was used to determine the cell viability in the circRHOT1 knockdown of non-small cell lung cancer cells (NSCLCs) and found that siRNA-mediated knockdown of circRHOT1 suppressed the cell viability in NSCLCs [124]. Cell proliferation, cell viability, and apoptosis were measured by cell counting kit-8 (CCK-8), MTT assay, and flow cytometry in HEK293 cells overexpressed with circ_0007059, and it was found that overexpression of circ_0007059 leads to increased cell proliferation, cell viability, and apoptosis [125]. Wang et al. [126] determined the effect of hsa_circ_0001038 depletion using CCK-8 and found that hsa_circ_0001038 depletion impairs the cell viability in HeLa cells. Sun et al. [127] determined the effect of circ-PVT1 knockdown in epithelial ovarian cancer cells using CCK-8 kit and Annexin V- FITC Apoptosis kit and found that Circ-PVT1 depletion leads to a decrease in cell proliferation but increases apoptosis.

## 7. Future Directions

Improvements in long-read sequencing, such as Oxford Nanopore and PacBio, with new technologies, help in the identification of full-length circRNA with more accuracy, avoiding errors. Technological advancements are proceeding to develop multiplexed imaging to visualize the different circRNA species and their interacting partners in the same cell. Further deep learning models using Artificial Intelligence may help in predicting the circRNA function and developing circRNA diagnostic markers, therapeutics, and vaccines more efficiently than traditional bioinformatics.

## 8. Conclusions

CircRNAs are a unique class of RNA molecules with high stability and diverse regulatory functions. Technological advances have significantly boosted the field of circRNA research, impacting areas like detection, functional analysis, and therapeutic development. This article presents different strategies or techniques used for each purpose, such as for RNA seq (RPAD, IsoCirc, and Sin SuperSeq); for detection (Northern blot and RT-qPCR); for visualization (in situ hybridization (circFISH, BaseScope, and padlock probe followed by RCA)); for circRNA biogenesis (RIP, CLIP, and PAR-CLIP); for circRNA-interacting partners (antisense-based pull-down assay and biotin-coupled pull-down assay); and for functional analysis (overexpression, siRNA- or shRNA-mediated knockdown, and CRISPR–Cas9 or CRISPR–Cas13-mediated knockout of circRNA). Overall, this article serves as a toolbox for people working in the field of circRNA (Table 2).

## Data Availability

No new data were created or analyzed in this study. Data sharing is not applicable to this article.
